# Supplementary material for: Visual Colorimetric Detection of Edible Oil Freshness for Peroxides Based on Nanocellulose
Source: Foods. 2023 May 5;12(9):1896. doi: 10.3390/foods12091896 (PMC10178133; doi:10.3390/foods12091896)
Supplement: Supplementary file 1 [file foods-12-01896-s001.zip › foods-2337237-supplementary.pdf]

Supporting information

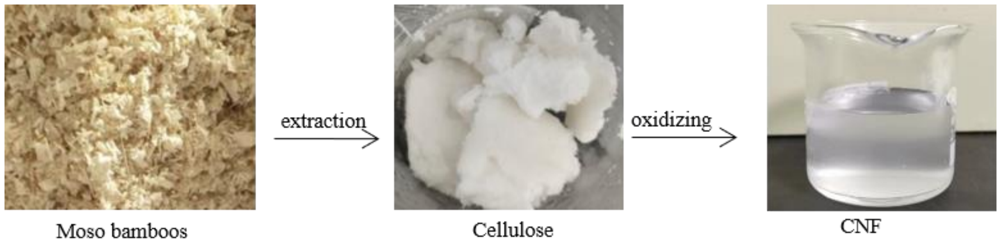

Figure S 1 Images of moso bamboos at different treatment stages.

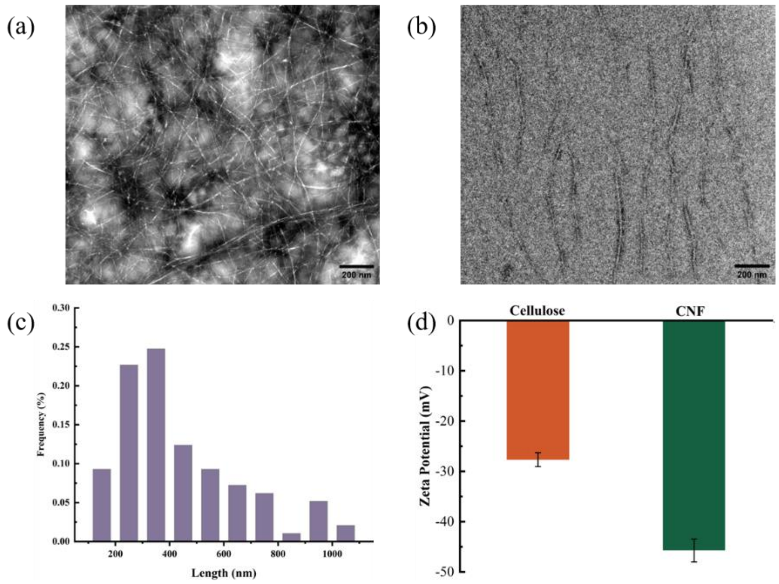

Figure S 2 TEM images of (a) bamboo cellulose and (b) CNF, (c) the corresponding statistic length distribution of CNF, (d) Zeta potential image of bamboo cellulose and CNF.

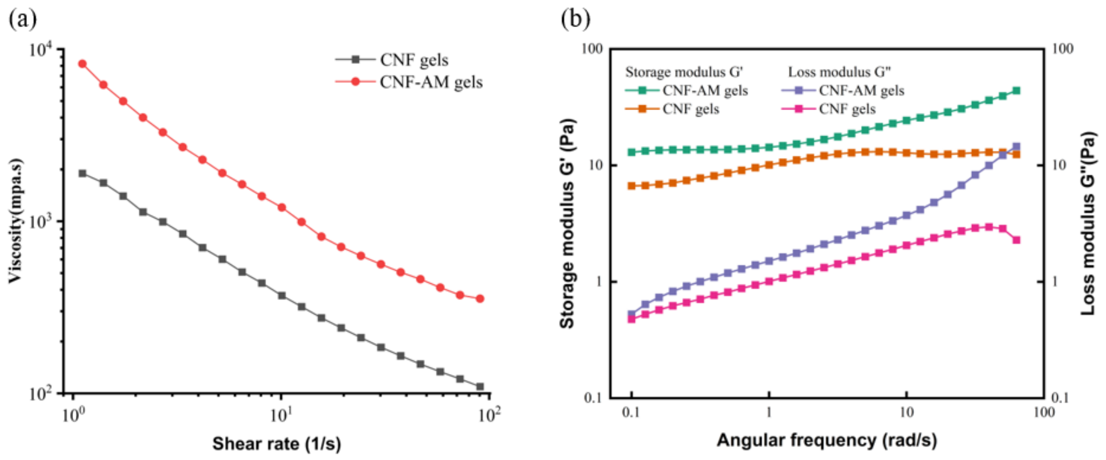

Figure S 3 (a) Compressive viscosity-shear rate curve of CNF gels and CNF-AM gels, (b) dynamic frequency sweep test of CNF gels and CNF-AM gels.

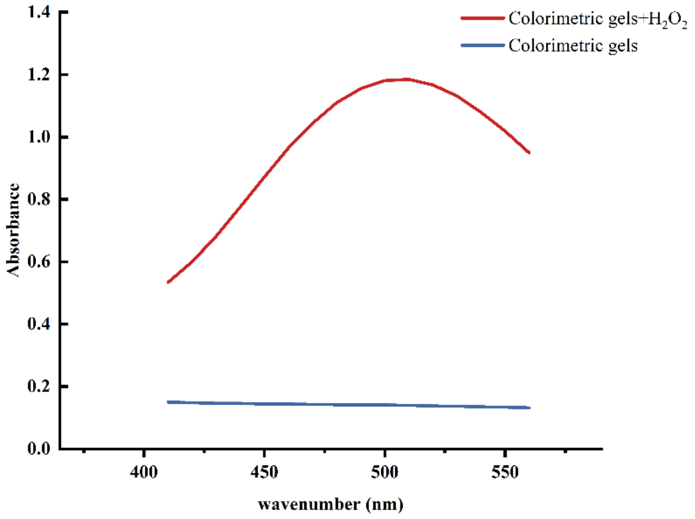

Figure S 4 UV–vis absorption spectra.

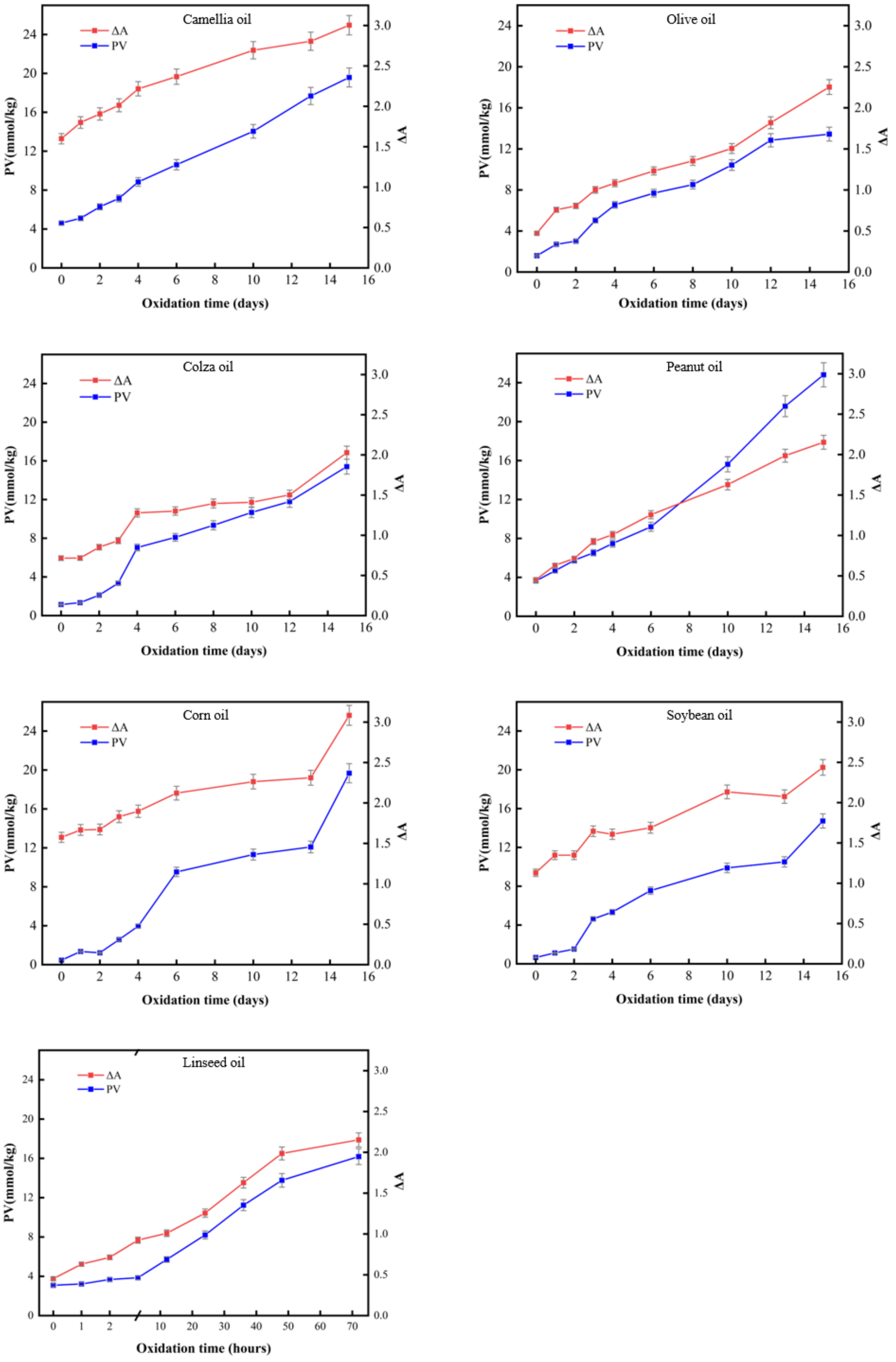

Figure S 5 The change of PV and ΔA of the edible oil held at different oxidation times.

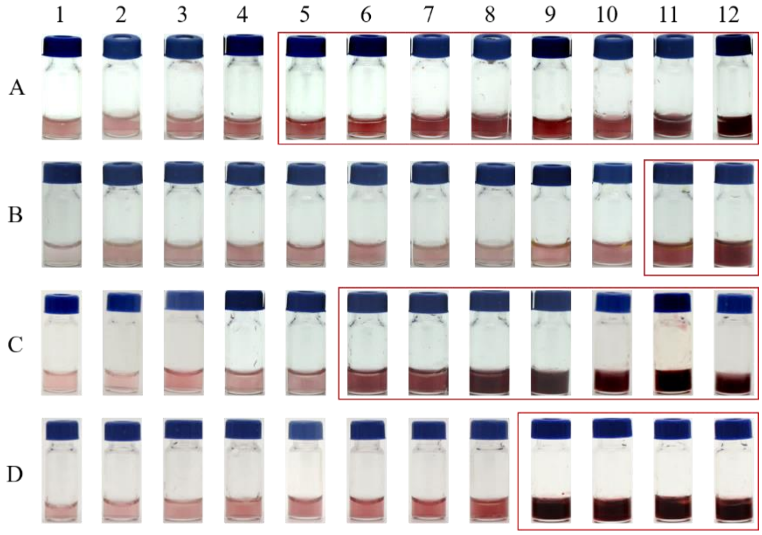

Figure S 6 Diagram of the reaction between peroxide responsive hydrogel and oil sample.

Table S 1 Comparison table of oil peroxide values (unit mmol/kg, n=3).

	1	2	3	4	5	6	7	8	9	10	11	12
A	3.09	3.21	3.67	3.85	5.71	7.19	8.20	11.24	13.76	12.39	17.29	31.28
B	1.14	1.50	2.23	1.93	3.55	6.35	2.45	3.97	7.36	7.95	10.08	18.89
C	1.32	1.17	3.40	3.22	3.22	8.83	9.89	11.18	12.02	15.00	15.19	18.70
D	0.92	1.55	2.46	3.76	3.97	6.12	7.36	7.95	10.08	15.90	18.89	19.85
